# Discordant *ALK* Status in Non-Small Cell Lung Carcinoma: A Detailed Reevaluation Comparing IHC, FISH, and NGS Analyses

**DOI:** 10.3390/ijms25158168

**Published:** 2024-07-26

**Authors:** Katarína Tobiášová, Martina Barthová, Ľuboslava Janáková, Katarína Lešková, Anna Farkašová, Dušan Loderer, Marián Grendár, Lukáš Plank

**Affiliations:** 1 Department of Pathological Anatomy, University Hospital Martin, Jessenius Faculty of Medicine, Comenius University, 036 01 Martin, Slovakia; tobiasova11@uniba.sk (K.T.);; 2 Martin’s Biopsy Center, Ltd., 036 01 Martin, Slovakia; 3 Biomedical Centre Martin—BioMed Martin, Jessenius Faculty of Medicine, Comenius University, 036 01 Martin, Slovakia

**Keywords:** ALK-rearranged NSCLC, ALK discordance, ALK protein expression, non-small cell lung carcinoma

## Abstract

*ALK* detection was performed on 2813 EGFR-unmutated NSCLC cases by simultaneous use of immunohistochemistry (VENTANA® anti-ALK D5F3, Roche Molecular Systems, Inc., Rotkreuz, Switzerland) and fluorescence in situ hybridization with the ALK break apart and the ALK/EML4 fusion probe (ZytoVision, Bremerhaven, Germany). A total of 33 cases were positive discordant (FISH-positive, IHC-negative) and 17 cases were negative discordant (FISH-negative, IHC-positive). This study’s aim was to reevaluate the methods used and compare discordant samples to positive concordant samples in order to ellucidate the differences. FISH signal variants were examined and compared. Positive discordant cases featured one pattern of *ALK* rearrangement in 41.4%, two patterns in 48.3%, and three patterns in 10.3% of analysed samples, with a higher variability of detected patterns and a higher number of *ALK* copy gains. Positive concordant cases displayed one pattern of rearrangement in 82%, two patterns in 17.8%, and three patterns in 0.6% of analysed samples. The association between number of patterns and concordance/discordance was statistically significant (*p* < 0.05). Eleven positive discordant and two negative concordant cases underwent NGS analysis, which resulted in identification of *ALK* fusion in one positive discordant and two negative discordant cases. Positive protein expression regardless of FISH result correlated more with a positive NGS result compared to samples with a positive FISH result with negative protein expression. FISH analysis was able to detect atypical or heterogenous patterns of rearrangement in a proportion of cases with negative protein expression, which may be associated with more extensive genetic alterations rather than true *ALK* rearrangement.

## 1. Introduction

Treatment of non-small cell lung carcinoma (NSCLC) has gone through significant changes in the span of last two decades. Based on presence of oncogenic mutations, two categories of NSCLC have been established—the “oncogene-addicted” and “non-oncogene-addicted” NSCLC [1], with separate guidelines for treatment.

One of the most frequent targetable genetic alterations in NSCLC is the rearrangement of the *ALK* gene, found in approximately 3–5% of all NSCLC cases [2]. ALK protein is a transmembrane protein with intracytoplasmic tyrosine-kinase domain, which upon activation triggers the downstream cell signaling cascades regulating proliferation, differentiation, and migration of cells. Oncogenic alteration of the *ALK* gene is usually caused by a chromosomal split in intron 19 of the *ALK* gene and the subsequent fusion of the tyrosine-kinase domain corresponding part to another partner gene, causing production of large quantities of the chimeric intracytoplasmic ALK protein [3] with aberrant activation independent of external stimuli. The second breaking point is usually localized within the *EML4* gene, leading to an intrachromosomal *ALK::EML4* inversion with currently more than 21 identified variants [4]; other fusions, such as *ALK::KIF5B*, *ALK::KLC1*, or *ALK::TPR*, occur less often, in approximately 5% of cases [5].

*ALK*-rearranged NSCLC responds well to small molecule inhibitors of receptors with tyrosine-kinase activity (TKIs). First-generation TKI crizotinib has a superior response rate compared to cytotoxic chemotherapy and displays significantly better overall survival (OS) as first-line therapy [6,7]. Second-generation CNS-penetrant ALK-TKIs, including ceritinib, alectinib, and brigatinib, show even longer progression-free survival (PFS) compared to crizotinib and therefore are currently preferred in first-line treatment [8,9,10,11].

*ALK* rearrangement detection has been performed mainly by immunohistochemistry (IHC) and in situ hybridization, nowadays with increasing demand for multiple gene analysis available through next-generation sequencing (NGS). Fluorescence in situ hybridization (FISH) allows detection of a split in the *ALK* gene on the chromosomal level; however, it is expensive, requires adequate quality of tissue, and expertise in interpretation of signal variants. *ALK::EML4* fusions tend to display a so-called short break pattern (less than two signal diameter), which can be interpreted as a false negative [12], though using the ALK/EML4 fusion probe mitigates this pitfall.

IHC analysis performed with more sensitive rabbit monoclonal antibodies (e.g., D5F3, Ventana) has become a valuable counterpart to FISH and is currently approved as a screening method for *ALK* rearrangement, even being acceptable as an equivalent alternative in the case of FISH unavailability or failure [1]. IHC is easier to interpret and cheaper; however, it cannot fully replace FISH as the method of choice [13,14,15], mainly due to the rare occurrence of discrepancies: some cases are either positive discordant (FISH+, IHC−) or negative discordant (FISH−, IHC+). This can be partially attributed to preanalytical steps [16], as well as post-translational changes and miscellaneous properties of the *ALK* protein produced by different rearrangement variants. A part of FISH negative cases can still be confirmed as *ALK* positive using another method [17]. IHC seems to correlate with NGS more and potentially is even more sensitive [18], although this can be attributed to technical challenges of FISH analysis. The simultaneous use of IHC and FISH is therefore still recommended [19,20], and whenever possible, discrepant cases should undergo analysis using NGS.

Discordance of ALK status has been a widely discussed topic, mainly due to clinical implications. Several case reports [21,22] and larger patient cohorts [23] conclude that ALK expression even with negative FISH result was associated with the desirable response to TKI treatment. Some published studies prove that patients with a positive discordant result may respond inadequately compared to negative discordant patients (62% response rate compared to 100% response rate) [24] or TKI treatment in positive discordant patients who are not confirmed as positive by NGS may be completely ineffective [25]. However, negative discordance (FISH−, IHC+) can predict worse overall survival [26] compared to concordant patients.

Our department performs the analysis of *ALK* status using IHC and FISH simultaneously, which over time naturally led to the accumulation of discordant cases. Our main concern was the reevaluation of methods used and finding possible explanations for the discrepancies. FISH analysis occasionally produced some unusual variants reports, leading us to the question whether the *ALK* discrepancies can be partially elucidated by comparing the FISH signal variants found in discordant cases to positive concordant samples. Furthermore, we aimed to validate these observations by comparing with NGS performed on a proportion of our cohort as a part of a pilot study.

## 2. Results

### 2.1. Concordance and Discordance between FISH and IHC

ALK detection using IHC and FISH simultaneously was successfully performed on 2683 samples. In 130 samples, one or both methods failed due to material limitations and were not further included into our study.

A total of 2444 samples were negative using both FISH and IHC, so-called negative concordant. A total of 184 cases were positive concordant; 38 cases were positive for *ALK* rearrangement using FISH, but negative for ALK protein expression and were labeled as positive discordant. In 9 of them, material quantity and quality were sufficient to repeat the IHC analysis. 5 cases displayed the so-called “dot-like” positivity and were reclassified as positive concordant. In 4 cases, the repeated IHC was still negative. The final number of positive concordant cases was 189, and the number of positive discordant cases was 33. A total of 17 cases were negative for *ALK* rearrangement with positive ALK protein expression and were labeled as negative discordant. Reevaluation of FISH did not reveal any false negatives.

Discordant cases altogether comprised 1.86% of all cases, with 1.23% being positive discordant and 0.63% being negative discordant. Out of all FISH-positive cases, 14.9% were positive discordant. Out of all FISH-negative cases, 0.7% were negative discordant. Sensitivity of FISH was calculated to be 91.7%, and specificity of FISH was 98.6%.

### 2.2. Demographic Distribution

The population group consisted of 1716 male patients and 967 female patients (Table 1), with age ranging from 23 to 91, with the mean of 65 years and median 66 years. NSCLC diagnosis occurred most frequently in patients in the sixth decade.

The overall frequency of *ALK* rearrangement was 5.5% in the male population, 13.1% in the female population, and 8.3% regardless of gender. *ALK* rearrangement was most prevalent in population of young patients, with peak incidence in female patients under 40 (7 out of 18 cases) and under 50 (15 out of 58 analyzed cases), as well as male patients under 50 years (22 out of 91 cases). With increasing age, the prevalence of *ALK* rearrangement became progressively lower but was more frequent in the female population in every age group. Negative discordant cases were found almost exclusively in the female population (14 out of 17 cases); positive discordant cases showed a slight male predominance (20 out of 33 cases).

### 2.3. Histopathology

In the positive discordant cohort, material limitations did not allow further specification than the “adenocarcinoma, NOS” category in 11 out of 33 cases (33.33%). The most frequently occurring types were solid predominant adenocarcinoma in four cases (12.12%), then G3 adenocarcinoma and metastatic adenocarcinoma in three cases (9.09%). Mucin production was especially noted in the report in 2 cases (6.06%), was not detected in 3 cases (9.09%), and was not specified in 28 cases (84.85%).

In the negative discordant cohort, the category “adenocarcinoma, NOS” was used in four cases (23.53%), equally in number as solid predominant adenocarcinoma (23.53%). Other subtypes did not occur particularly frequently. Mucin production was specially noted in the report in 1 case (5.88%), was not detected in 2 cases (11.76%) and was not specified in 14 cases (82.35%).

In the positive concordant cohort, “adenocarcinoma, NOS” was used in 57 cases (30.16%), followed equally by acinar predominant, solid predominant, and metastatic adenocarcinoma, each comprising 21 cases (11.11%). “Adenocarcinoma, NOS, mucin producing”, and mucinous adenocarcinoma accounted for eight cases each (4.23%), followed by cases classified as micropapillary predominant adenocarcinoma and G3 adenocarcinoma, with six cases for each category (3.17%), and signet ring cell adenocarcinoma with four cases (2.12%). Mucin production was noted in at least one component in 38 cases (20.10%), not detected in 6 cases (3.17%), and not specified in 145 cases (76.72%) (Table 2).

### 2.4. Comparison of ALK Copy Number Gains in ALK Concordant and Discordant Samples

The positive discordant cohort had 3 or less *ALK* copies in 12 out of 33 cases (36.36%), 4 to 6 *ALK* copies in 8 cases (24.24%), 7 to 9 *ALK* copies in 6 cases (18.18%), and 10+ *ALK* copies in 7 cases (21.21%). The negative discordant cohort featured three or less *ALK* copies in 11 cases (64.7%), 4 to 6 *ALK* copies in 2 cases (11.76%), 7 to 9 *ALK* copies in 1 case (5.88%), and 10+ *ALK* copies in 3 cases (17.65%). The positive concordant cohort displayed 3 or less *ALK* copies in 129 cases (68.25%), 4 to 6 *ALK* copies in 40 cases (21.16%), 7 to 9 *ALK* copies in 19 cases (10.05%), and 10+ *ALK* copies in 1 case (0.53%) (Table 3).

### 2.5. FISH Pattern Distribution in Positive Discordant and Positive Concordant Samples

A total of 197 samples previously tested with ALK break apart probe were analyzed with ALK/EML4 fusion probe and classified according to their predominant pattern of rearrangement as described in Section 4. The cohort consisted of 29 positive discordant samples and 168 positive concordant samples.

#### 2.5.1. Positive Discordant Samples

In 13 samples (44.8%), the positive result was a combination of several signal variants each below 15%. In five of them, it was possible after elimination of sectioning artifacts to identify one pattern of rearrangement (all inversion). In seven samples, two patterns were identified, and in one sample, three patterns were found. In 16 samples (51.8%), at least one pattern of rearrangement reached the cut off. Seven of them were evaluated as containing one rearrangement pattern (five cases of inversion, two cases of 5′ deletion); in seven samples, two patterns of rearrangement were identified, and in two samples, three patterns of rearrangement were identified (Table 4).

One pattern was overall identified in 12 out of 29 samples (41.4%), two patterns were found in 14 samples (48.3%) and three patterns in 3 samples (10.3%). Inversion was identified in 10 out of 12 samples with single rearrangement pattern (83.3%). Another single rearrangement pattern was 5′ deletion, in 2 cases (16.7%). Interstitial deletion and translocation did not occur as single identified patterns, only in combinations.

**Figure 1:**
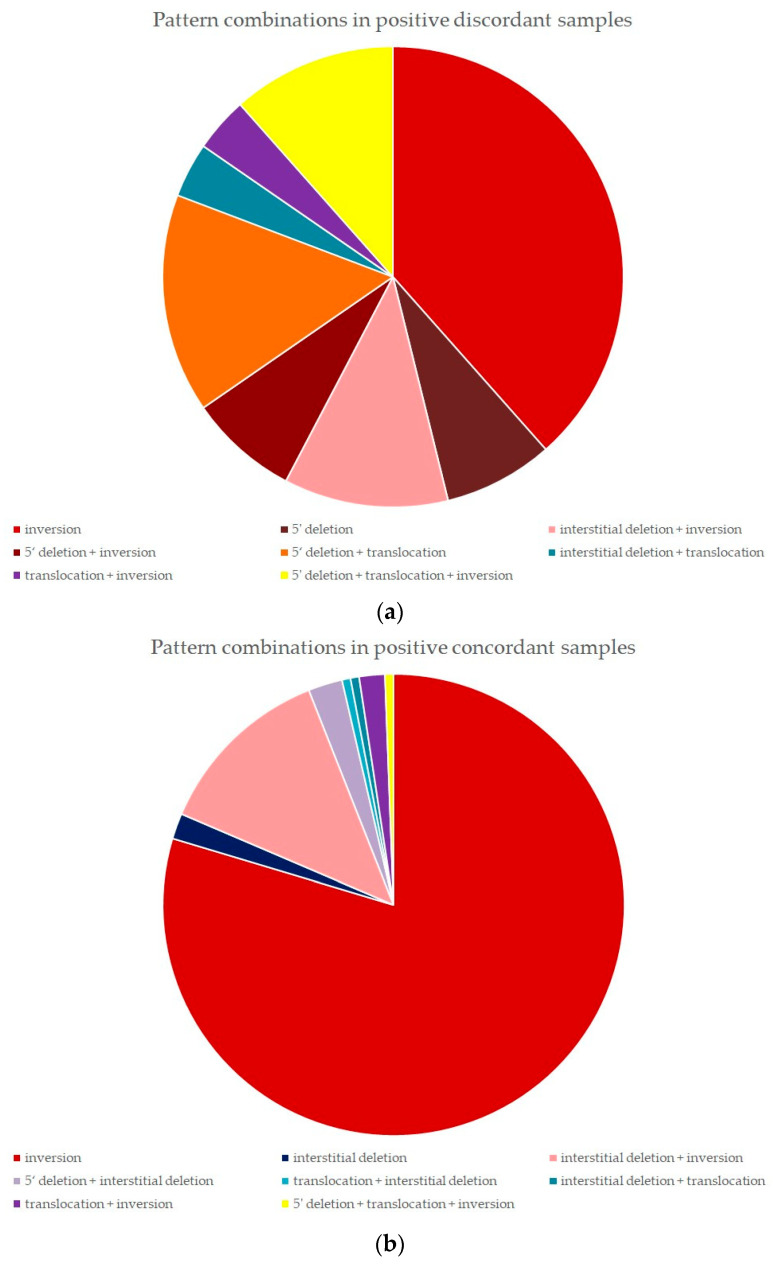
(**a**) Pattern combinations identified in positive discordant samples. (**b**) Pattern combinations identified in positive concordant samples.

Out of two pattern combinations, variants featuring mainly 5′ deletion were present in 6 out of 14 samples (42.8%), interstitial deletion as the predominant pattern was found in 4 samples (28.6%), inversion in 3 samples (21.4%), and translocation in 1 sample (7.1%). Three samples with three rearrangement patterns all contained 5′ deletion combined with translocation and inversion (in that order of percentage) (Figure 1).

#### 2.5.2. Positive Concordant Samples

In two samples (1.2%), the positive result was a combination of several signal variants each below 15%, which were, however, identified as resulting from inversion. In 166 cases (98.8%), at least one signal variant met or exceeded the 15% cut-off. A single pattern of rearrangement was overall found in 137 out of 168 samples (81.5%). A total of 30 samples (17.8%) were classified as containing two patterns. One sample featured three rearrangement patterns (0.6%) (Table 4). Samples with one pattern identified featured almost exclusively inversion in 133 samples. Four samples (3%) displayed the interstitial deletion pattern. Translocation and 5′ end deletion did not occur as a single pattern, only in combinations.

A total of 30 samples had a combination of two patterns, with the most frequent one being interstitial deletion and inversion in 21 samples (70%), followed by 5′ deletion coupled with interstitial deletion in four samples (13.3%), as well as translocation combined with inversion in three samples (10%). Interstitial deletion combined with translocation, and the reverse order variant translocation combined with interstitial deletion, were found in one case (3.3%) each. One sample with three identified patterns featured 5′ deletion, translocation, and inversion (Figure 1).

#### 2.5.3. Statistical Analysis of Pattern Distribution

There was a statistically significant association (*p*-value 0.0000055) between the type of sample (concordant/discordant) and variant of FISH signal (three patterns). Using the standard calibration of Cramer’s V for 2 degrees of freedom, the association may be judged strong (Cramer’s V is 0.37, with 95% confidence interval (0.24, 0.51)). Pattern frequency did not show a statistically significant association between the type of sample (concordant/discordant) (Supplementary Materials File S1).

### 2.6. NGS Analysis

NGS analysis was successfully performed on 75 samples: 58 negative concordant, 4 positive concordant, 11 positive discordant, and 2 negative discordant cases. NGS did not identify *ALK* fusion in any of the negative discordant cases (other alterations, not listed in this publication, were found). NGS identified *ALK::EML4* fusion in three positive cases and cases and *HIP::ALK* fusion in one positive concordant case. In 10 positive discordant cases, *ALK* fusion was not detected, and one case resulted in identification of *ALK::EML4* fusion transcript. In two negative discordant samples, NGS detected *ALK* fusion transcripts (*ALK::EML4* and *ALK::CSFT3*) (Table 5).

## 3. Discussion

Our main concern was to reevaluate the results of the applied tests of samples with discordant results and reevaluate correct interpretations of all the analyses. In the second step, comparison between methods was performed. This process revealed striking differences between positive discordant and positive concordant cases.

FISH patterns of rearrangement in the positive concordant cohort almost always (98.8%) reached levels above the cut-off value and consisted mainly of one common pattern of rearrangement (81.5%) identified in all positive nuclei. The positive discordant cohort partially (44.8%) consisted of cases where the positive result was reached by a combination of signal variants below the 15% cut-off. Higher variability in number of rearrangement patterns was also recorded (41.4% cases had one rearrangement pattern, two patterns were found in 48.3% samples and three patterns in 10.3% of samples), which was proven to be statistically significant. Heterogeneity of *ALK* rearrangement in positive discordant samples might be therefore responsible for the lack of ALK protein expression, and the ALK status should be evaluated by another method.

The positive concordant group showed high predominance of tumor samples featuring the classic *ALK::EML4* inversion, either as the single pattern (97% of samples with one pattern identified and 79.1% of all positive concordant samples) or very frequently coupled with interstitial deletion (70% of samples with two patterns identified). In contrast, inversion was the most frequently occurring single pattern in positive discordant samples as well (83.3% of cases with one pattern identified), but overall, it comprised only approximately over a third of all positive discordant cases.

5′ deletion never occurred on its own in the positive discordant group, but it represented the main rearrangement pattern in 5 out of 31 samples with two or three identified patterns (16.1%). In the positive discordant group, it was found as the only pattern in two cases, and as the main pattern in 9 out of 17 (52.9%) samples with two or more identified patterns of rearrangement.

The FISH result featuring the isolated 5‘ orange signal with negative ALK protein expression may signify a false positive case, as was examined in a study by Gao et al. [27], in which IHC-negative patients with this FISH signal variant did not fit into the typical profile of *ALK*-positive patients, with older age at diagnosis and more extensive smoking history. The FISH result was estimated to be caused by structural variants affecting the 5′ binding site, associated with higher genomic instability of the tumor typical for prolonged smoking; therefore, it likely did not represent true *ALK* rearrangement. Clavé et al. [28] describe two IHC-negative cases with a predominant 5′ deletion signal that tested negative for *ALK* fusion using NGS, with the recommendation to always retest this particular rearrangement variant. A similar finding was reported by Dacic et al. [29], where NGS confirmed *ALK* fusion only in 4 out of 10 cases with 5′ end deletion pattern only, while all showed IHC negativity.

IHC-positive cases, regardless of FISH status, tended to have fewer *ALK* CNG, as seen in 129 (68.25%) positive concordant cases, compared to 12 (36.36%) positive discordant cases. Multiple copies of *ALK* can be caused by *ALK* amplification or polysomy, but neither variant shows any clinical significance for indication of TKI treatment [30]. Increased CNG likely comes from early genetic events triggered by cigarette smoking, independent of true *ALK* rearrangement, and is associated with genomic instability and more aggressive biological behavior, as well as potential higher sensitivity to chemotherapy [31,32], and also including one of the mechanisms of crizotinib resistance both in vitro and in patients progressing on TKI treatment [33,34]. Lower CNG in IHC-positive cases may indicate a more stable genomic profile compared to higher heterogeneity of positive discordant cases.

Cases with a notable presence of multiple components were described as such in the FISH report, and in our cohort were found predominantly in the positive discordant group, including 4 out of 13 positive discordant samples re-tested with NGS (cases 6, 9, 10, and 11). In these cases, either the *ALK* rearranged part represented only a small component of the overall tumor mass (cases 9 and 11), or these tumors contained two different components (adenocarcinoma and squamous cell carcinoma for case 6, well-differentiated and poorly differentiated component in case 10). The pattern of rearrangement included 5‘ end deletion (case 9), inversion (case 6, only adenocarcinoma component) and translocation with inversion (poorly differentiated area of case 10). A small part of the tumor cells may display alterations of the *ALK* locus, but its clinical significance and targetability by TKIs remains questionable. The patients’ age in this subgroup of four patients (two male patients aged 63 and 78 and two female patients aged 70 and 79) also fell outside of the typical profile of ALK-positive patients; however, this parameter cannot be reliably used to determine *ALK* status. It is worth noting, though, that NGS analysis did not detect *ALK* fusion in any of these cases.

Out of 11 positive discordant cases tested with NGS, only one case (Case 3, Table 5) revealed by NGS the *ALK::EML4* fusion transcript, and in all the other the NGS did not demonstrate *ALK* fusion. In comparison, *ALK* fusion was identified in two out of two negative discordant cases tested by NGS. One case (Case 12, Table 4) featured predominantly an unusual 3′ deletion pattern in 74% of evaluated nuclei. The 3′ end of the hybridization probe binds to the tyrosine-kinase domain of the *ALK* gene necessary for activation of signal pathways; therefore, this alteration has uncertain clinical implications. Although the *ALK* locus was obviously altered in this sample and ALK protein expression was detected, it could not be classified as *ALK* positive under valid guidelines. However, NGS analysis revealed *ALK::EML4* fusion transcript and *KLF7::EML4* fusion as an additional finding.

NGS analysis in our cohort corresponded more with positive protein IHC expression than with FISH results, and similar results were also reported by Lin et al. [18]. We might agree with the authors that it does not imply FISH to be less reliable, but that it rather points out the technical challenges of interpreting FISH and higher requirements for tissue quality to be eligible for testing. Our own experience in detailed analysis of all occurring signal variants offers a plausible explanation: FISH might be able to detect alterations in *ALK* locus associated with more extensive mutational profile. It is not entirely correct to consider these FISH results as false positives, if the method is performed adequately and interpreted correctly, but notable heterogeneity in detected rearrangement patterns, along with increased *ALK* CNGs in cases with negative ALK protein expression, can indicate more severe genomic alterations in the analyzed sample. All such cases should be therefore verified using NGS.

## 4. Materials and Methods

A team composed of the Pathology Department of JFM CU and UH Martin and Martin’s Biopsy Center, Ltd., performed the detection of ALK protein expression using D5F3 antibody in 2813 NSCLC specimens, with all EGFR unmutated as examined by RT-PCR, as a part of the standard diagnosis algorithm. FFPE samples of either primary metastatic NSCLC were sent from other departments to undergo further molecular analysis to detect relevant predictive biomarkers of either targeted treatment and/or immunotherapy, including *ALK*, *ROS-1*, *MET*, *RET,* and *NTRK1/2/3* alterations and PDL-1 status. *ALK* rearrangement was tested by two standard methods: on the level of protein expression by IHC, and on the cytogenetic level using FISH. Discordant cases were carefully evaluated, and a small fraction of cases was additionally analyzed using NGS as a part of a pilot study.

### 4.1. Immunohistochemistry

Immunohistochemical analysis of ALK protein expression was performed on 2813 of 3–4 µm thick FFPE sections using monoclonal antibody VENTANA anti-ALK (clone D5F3, Roche Molecular Systems, Inc., Rotkreuz, Switzerland) on the BenchMark GX platform (Roche Molecular Systems, Inc., Rotkreuz, Switzerland). The analysis was realized according to the protocol recommended by the manufacturer. Sections were kept in the thermostat at 37 °C all night and then were placed into BenchMark GX. After staining, sections were rinsed in running water for 10 min, dehydrated with graded alcohol and xylene, and coverslipped.

IHC quality control was performed through QuiP (Qualitatssicherungs-Initiative Pathologie GmbH, Berlin, Germany). Internal control was a part of every analyzed slide, using tissue from another *ALK*-positive case, which was exchanged regularly when the block aged. The whole slide was stained, but only the tumor area was evaluated for ALK protein expression. The outcomes were evaluated semiquantitatively according to the following criteria: the presence of strong diffuse granular cytoplasmic staining (2+, 3+). In the reevaluation stage, the so-called dot-like positivity was also labelled as positive. No staining, weak cytoplasmic staining (1+), or so-called stippled pattern were all interpreted as negative.

### 4.2. Fluorescence In Situ Hybridization

#### FISH Analysis

FISH analysis was performed on 3–4 µm thick formalin-fixed paraffin-embedded (FFPE) tissue sections using ZytoLight^®^SPEC ALK Dual Color Break Apart Probe (ZytoVision, GmbH, Bremerhaven, Germany). FFPE specimens were processed manually using a ZytoLight^®^FISH-Tissue Implementation Kit according to the manufacturer’s instructions. Results were analyzed using a fluorescence BX61 microscope (Olympus, Tokyo, Japan) with the support of the software Lucia FISH v.3.1 (Laboratory Imaging, Prague, Czech Republic).

*ALK* rearrangement was scored according to the valid guidelines [35]. In all cases with sufficient tumor cell count, 100 nuclei were enumerated. If material was critically limited, then the evaluated tumor cell count was lower than 100 but had to include minimally 50 cells. Cases with less than 50 cells were excluded from our study.

The presence of two isolated signals (3′ orange signal and 5′ green signal) at least two-signal diameter apart and/or a single 3′-isolated orange signal were marked as positive, demonstrating a split in the structure of *ALK* gene. An orange/green fusion signal indicated an intact *ALK* locus, andtherefore it was interpreted as negative. Specimens with ≥15% of positive cells were classified as positive for *ALK* rearrangement, while cases with <15% were classified as negative. All positive and some equivocal samples displaying the so-called short break pattern (less than two-signal diameter) were retested again using the ZytoLight ^®^SPEC ALK/EML4 TriCheck™Probe. *ALK* rearrangement was scored according to the manufacturer’s instructions [36].

*ALK* copy number gain (CNG) was evaluated using the ALK break apart probe, without distinction between polysomy and amplification, displayed as cells with multiple copies of an intact *ALK* locus (multiple fusion signals in a single nucleus). Four categories were established: “three or less *ALK* CNGs”, “4–6 *ALK* CNGs”, “7–9 *ALK* CNGs”, and “10+ *ALK* CNGs”.

Positive discordant and positive concordant cases retested with ALK/EML4 fusion probe were compared in terms of FISH rearrangement variants.

Translocation with other than the *EML4* partner was indicated by the presence of (a) isolated 3′ orange signal and isolated 5′ green signal co-localizing with blue signal (O/GB); (b) isolated 3′ orange signal and isolated 5′ green signal (O/G); or (c) isolated 3′ orange signal, isolated 5′ green signal, and isolated blue signal (O/G/B).

**Figure 2:**
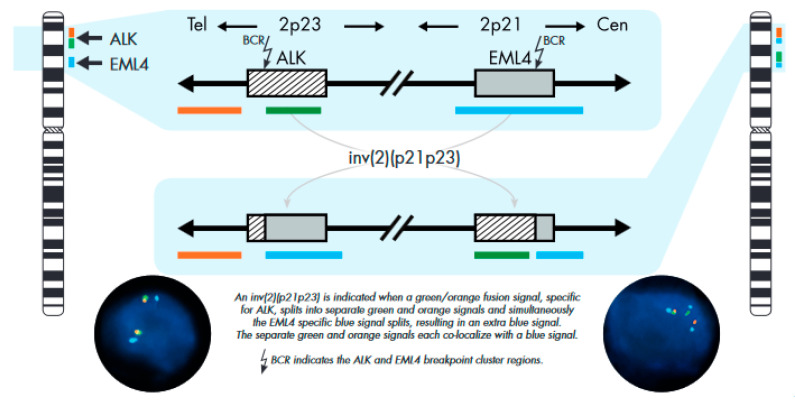
ZytoLight ^®^ SPEC ALK/EML4 TriCheck™ Probe Design, reprinted with permission from Schildhaus HU et al. [36] Signal interpretation guide, 2016. © 2024 ZYTOVISION GmbH.

ALK/EML4 inversion pattern included one or more of the following variants: (a) isolated 3′ orange signal co-localizing with blue signal and isolated 5′ green signal co-localizing with blue signal (Figure 2) (OB/GB); (b) isolated 3′ orange signal co-localizing with blue signal and isolated 5′ green signal (OB/G); (c) isolated 3′orange signal co-localizing with blue signal, isolated blue signal, and isolated 5′ green signal (OB/B/G); or (d) inversion with partial *ALK* gene deletion, represented by isolated 3′ orange signal co-localizing with blue signal and another isolated blue signal (OB/B).

The 5′ end deletion of the *ALK* gene was represented by two variants: (a) isolated 3′ orange signal, loss of 5′ green signal (O), or (b) isolated 3′ orange signal, isolated blue signal, loss of 5′green signal (O/B).

Interstitial deletion of the *ALK* gene was indicated by the presence of an isolated 3′ orange signal co-localizing with a blue signal (OB).

**Figure 3:**
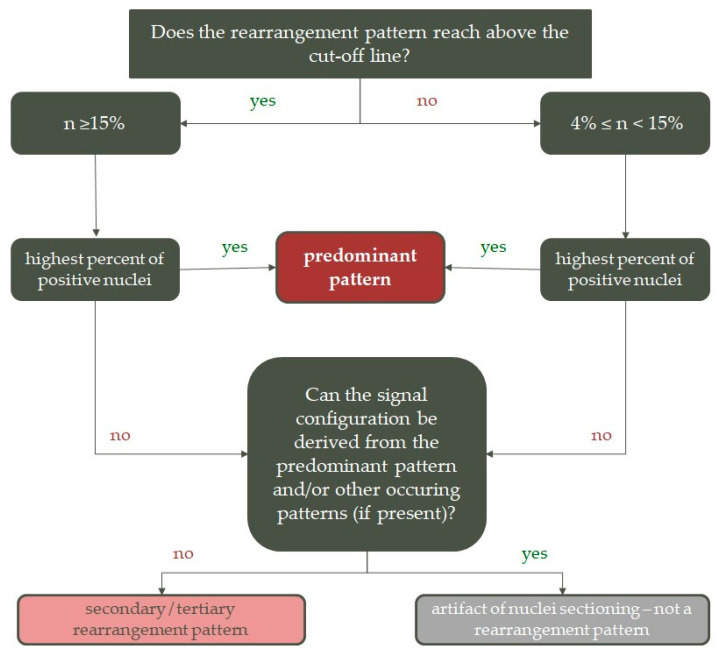
Classification of FISH patterns in samples analyzed with ALK/EML4 fusion probe.

When analyzing samples, either at least one signal combination on its own reached above the 15% cut-off, or the positive result was composed of several rearrangement variants, each with less than 15% of positivity, but at the very least found in 4% of rearranged nuclei (Figure 3).

The predominant pattern of rearrangement was the signal combination with the highest percent of positivity in rearranged cells. All other identified patterns were then evaluated based on whether or not they could have been derived from the predominant pattern. If the minority pattern was less complex, featured less signals, or its configuration was a part of the predominant pattern, it was not considered a secondary pattern, but most likely a result of tissue sectioning. If the minority pattern was found in at least 4% of rearranged nuclei, was more complex, featured more fluorescent signals, or its structure could not have been derived from the predominant pattern, it was considered a secondary (or tertiary) pattern of rearrangement. Details and examples of this assortment can be found in Supplementary Materials File S2.

### 4.3. Next-Generation Sequencing

Sequencing was performed with the panel TruSight Oncology 500 Assay (Illumina, San Diego, CA, USA), enabling simultaneous analysis of DNA and RNA extracted from the same sample. This assay provides detection of SNVs and indels in 523 cancer-related genes, CNVs of 69 genes, gene fusions and splice variant of 55 driver genes, and immunotherapy biomarkers that rely on the analysis of multiple genomic loci such as tumor mutational burden (TMB) and microsatellite instability (MSI).

Tissue was sectioned into 4 to 6 10 μm thick slices and deparaffinized using Qiagen solution. Isolated DNA was treated with RNAse A to eliminate contamination from RNA; similarly, RNA was treated with DNAse A to ensure high purity of nucleic acids.

DNA quantity was measured with a Qubit dsDNA BR kit (Invitrogen (Carlsbad, CA, USA), Thermo Fisher Scientific (Waltham, MA, USA)) on the Qubit 4 Flurometer platform, (Thermo Fisher Scientific, Waltham, MA, USA). The minimal required amount of DNA was 80 ng/sample; samples that did not meet this criterion were excluded. Quality control was performed with Infinium FFPE QC kit (Illumina, USA) according to the manufacturer’s guidelines.

RNA quality was measured with an RNA 6000 Nano kit (Agilent, Santa Clara, CA, USA), and the level of DV200 was assessed. This number corresponded to the percentage of RNA fragments with length greater than 200 bp. DV200 < 30% signified a high level of RNA degradation not suitable for NGS analysis, and these samples were excluded. Samples with a concentration of RNA higher than 10 ng/μL and DV200 > 30% were used for the preparation of libraries.

According to the manufacturer’s instruction for Illumina TruSight 500 Oncology Assay, 16 libraries were manually processed, containing 8 DNA and 8 cDNA. Finally, libraries with concentration >3 ng/µL were normalized, pooled, and loaded on a NextSeq 550Dx (Illumina, San Diego, CA, USA). The paired-end sequencing runs (2 × 101 bp) were performed in high output mode with a High Output Kit v2.5 (300 Cycles). Output from NGS was subjected to bioinformatic processing by Illumina LocalApp ver. 2.2.0. There is no cutoff on variant allele frequency (VAF) in the LocalApp pipeline. The cutoff there is set in a dynamic manner, depending on loci and sample quality, and it can be as low as 2–3 mutant reads. In addition, there are two quality scores: AQ (variant artifact adjusted quality score; *p*-value against baseline samples) and LQ (likelihood ratio quality score; sample specific *p*-value). Point mutations were identified as one nucleotide, insertions as the length of 18 bp, and deletion between 10 and 25 bp.

For the fusion calling, the unique reads were used. In order for a fusion to be called with high confidence, at least 5 unique reads were needed. Concerning the copy number variation (CNV), the TSO500 panel allows for CNV calling in 59 genes. Each gene had a different noise profile. For this reason, each gene had specific thresholds for calling amplifications and deletions. Samples with a tumor content above 20% were analyzed in our study (for amplification, it means 14 copies at the 20% tumor purity).

The results of bionformatic processing were afterwards annotated by the PierianDX Clinical Genomics Workspace (CGW). The annotation involves, among other things, ranking of the called SNVs, CNVs, and fusions into tiers, according to standards and guidelines for the interpretation and reporting of sequence variants in cancer: a joint consensus recommendation of the Association for Molecular Pathology, American Society of Clinical Oncology, and College of American Pathologists [37].

### 4.4. Comparison of IHC and FISH

Sensitivity of FISH was calculated as the number of positive concordant results divided by positive concordant and FISH-negative discordant cases. Specificity was calculated as the number of negative concordant results divided by negative discordant and FISH-positive discordant results. Concordance between *ALK* rearrangement and ALK protein expression was performed. Demographic characteristics of population groups were evaluated, and differences in *ALK* rearrangement among population groups were assessed.

FISH results of positive concordant and FISH-positive discordant cases were further sorted according to patterns detectable using ALK/EML4 fusion probe. The total number of detected patterns was recorded in FISH-positive concordant and FISH-positive discordant cases, and the predominant pattern of rearrangement was identified. If the sample contained more patterns, their combination was also documented. *ALK* CNG was compared between positive concordant and all discordant cases.

### 4.5. Statistical Analysis

Data were explored and analyzed in R [1], ver. 4.0.5, with the aid of libraries [38,39,40,41]. Contingency tables were visualized by mosaicplot. The null hypothesis of no association between two factors was tested by Fisher’s exact test. Effect size was measured by Cramer’s V, and its uncertainty was quantified by a 95% double-sided chi-squared confidence interval. Where there was row of 0 frequencies in a contingency table, the Laplace smoothing was applied to the table prior to hypothesis testing.

## 5. Conclusions

Our department performed simultaneous detection of *ALK* rearrangement using both FISH and IHC, which aided in more reliable detection of *ALK* rearrangement in case of failure of one of the methods. FISH results are interpreted with detailed description of all occurring patterns and CNGs, and a thorough look at the ALK rearrangement variants might contribute to the explanation of the lacking protein expression in positive discordant cases.

Positive concordant samples tended to display the classic inversion and also show less variability in detected variants, suggesting that the genomic profile of the tumor population is more stable compared to the IHC-negative cases, which correlates with the typical demographic group of younger, non-smoker patients. Positive discordant cases were associated with a higher variability of detected rearrangement variants, as well as with the 5′ deletion pattern. FISH-positive cases without ALK protein expression displaying one of these characteristics should be promptly verified with another method.

NGS results seem to show a correlation with ALK-protein-positive expression in our limited cohort. In conclusion, the simultaneous use of IHC and FISH, as is the standard in many departments including ours, allows for the selection of the highest number of patients eligible for targeted treatment. Further implementation of NGS is crucial in improving diagnosis and detection of other predictive biomarkers and indicating the best possible treatment for NSCLC patients.

## Figures and Tables

**Table 1:** *ALK* rearrangement detection and *ALK* discordance among population groups.

Age	Total	Positive FISH	Negative FISH	Discordant Cases	
				FISH−, IHC+	FISH+, IHC−
20–29	6	2	4	1	0
20–29 men	2	0	2	0	0
20–29 women	4	2	2	1	0
30–39	28	9	19	0	0
30–39 men	14	4	10	0	0
30–39 women	14	5	9	0	0
40–49	133	33	100	1	2
40–49 men	75	18	57	0	2
40–49 women	58	15	43	1	0
50–59	487	55	432	1	7
50–59 men	296	26	270	0	6
50–59 women	191	29	162	1	1
60–69	1170	76	1094	6	18
60–69 men	737	29	708	1	10
60–69 women	433	47	386	5	8
70–79	743	41	702	7	6
70–79 men	520	16	504	2	2
70–79 women	223	25	198	5	4
80–89	114	6	108	1	0
80–89 men	71	2	69	0	0
80–89 women	43	4	39	1	0
90–99	2	0	2	0	0
90–99 men	1	0	1	0	0
90–99 women	1	0	1	0	0
total	2683	222	2461	17	33
men	1716	95	1621	3	20
women	967	127	840	14	13

**Table 2:** Histopathological data as provided by sending departments (no second look option).

Histological Type	Total (*n* = 239)	Positive Discordant (*n* = 33)	Negative Discordant (*n* = 17)	Positive Concordant (*n* = 189)
Squamous cell carcinoma	4 (1.67%)	2 (6.06%)	2 (11.76%)	0 (0.00%)
Minimally invasive adenocarcinoma	2 (0.83%)	1 (3.03%)	1 (5.88%)	0 (0.00%)
Adenocarcinoma, NOS	72 (30.12%)	11 (33.33%)	4 (23.53%)	57 (30.16%)
Adenocarcinoma, NOS, mucin not detected	6 (2.51%)	1 (3.03%)	0 (0.00%)	5 (2.65%)
Adenocarcinoma, NOS, mucin producing	8 (3.35%)	0 (0.00%)	0 (0.00%)	8 (4.23%)
Lepidic predominant adenocarcinoma	3 (1.26%)	1 (3.03%)	0 (0.00%)	2 (1.06%)
Acinar predominant adenocarcinoma	24 (10.04%)	2 (6.06%)	1 (5.88%)	21 (11.11%)
Papillary predominant adenocarcinoma	12 (5.02%)	0 (0.00%)	1 (5.88%)	11 (5.82%)
Solid predominant adenocarcinoma	29 (12.13%)	4 (12.12%)	4 (23.53%)	21 (11.11%)
Micropapillary predominant adenocarcinoma	6 (2.51%)	0 (0.00%)	0 (0.00%)	6 (3.17%)
Signet ring cell adenocarcinoma	4 (1.67%)	0 (0.00%)	0 (0.00%)	4 (2.12%)
Mucinous adenocarcinoma	9 (3.77%)	1 (3.03%)	0 (0.00%)	8 (4.23%)
Clear cell adenocarcinoma	1 (0.42%)	1 (3.03%)	0 (0.00%)	0 (0.00%)
G3 adenocarcinoma, growth patterns not specified	9 (3.77%)	3 (9.09%)	0 (0.00%)	6 (3.17%)
G2 adenocarcinoma, growth patterns not specified	1 (0.42%)	0 (0.00%)	0 (0.00%)	1 (0.53%)
Pleomorphic carcinoma	1 (0.42%)	1 (3.03%)	0 (0.00%)	0 (0.00%)
Sarcomatoid carcinoma	1 (0.42%)	0 (0.00%)	0 (0.00%)	1 (0.53%)
Adenosquamous carcinoma	3 (1.26%)	1 (3.03%)	0 (0.00%)	2 (1.06%)
Combined NEC and adenocarcinoma	2 (0.83%)	1 (3.03%)	0 (0.00%)	1 (0.53%)
NSCLC, NOS	3 (1.26%)	0 (0.00%)	0 (0.00%)	3 (1.59%)
Metastatic adenocarcinoma	26 (10.88%)	3 (9.09%)	2 (11.76%)	21 (11.11%)
Data unavailable	13 (5.44%)	0 (0.00%)	2 (11.76%)	11 (5.82%)
Mucin production in one or more components	41 (17.01%)	2 (6.06%)	1 (5.88%)	38 (20.10%)
Mucin not detected	11 (4.56%)	3 (9.09%)	2 (11.76%)	6 (3.17%)
Not specified	187 (77.59%)	28 (84.85%)	14 (82.35%)	145 (76.72%)

**Table 3:** Comparison of *ALK* copy number gains in all discordant vs. positive concordant samples.

	Positive Discordant (*n* = 33)	Negative Discordant (*n* = 17)	Positive Concordant (*n* = 189)
3 or less *ALK* CNGs	12 (36.36%)	11 (64.70%)	129 (68.25%)
4–6 *ALK* CNGs	8 (24.24%)	2 (11.76%)	40 (21.16%)
7–9 *ALK* CNGs	6 (18.18%)	1 (5.88%)	19 (10.05%)
10+ *ALK* CNGs	7 (21.21%)	3 (17.65%)	1 (0.53%)

**Table 4:** Pattern distribution in positive concordant and positive discordant samples.

	Patterns <15%	At Least 1 Pattern ≥15%	Total
Discordant samples (IHC−, FISH+)	13	16	29
1 pattern identified	5	7	12
Inversion	5	5	10
Interstitial deletion	0	0	0
Translocation	0	0	0
5′ deletion	0	2	2
2 patterns identified	7	7	14
Interstitial deletion + inversion	0	3	3
5′ deletion + inversion	2	0	2
5′ deletion + translocation	0	4	4
Interstitial deletion + translocation	1	0	1
Translocation + inversion	1	0	1
Inversion + translocation	3	0	3
3 patterns identified	1	2	3
5′ deletion + translocation + inversion	1	2	3
Concordant samples (IHC+, FISH+)	2	166	168
1 pattern identified	2	135	137
Inversion	2	131	133
Interstitial deletion	0	3	3
Translocation	0	0	0
5′ deletion	0	0	0
2 patterns identified	0	30	30
Interstitial deletion + inversion	0	21	21
5′ deletion + interstitial deletion	0	4	4
Translocation + interstitial deletion	0	1	1
Interstitial deletion + translocation	0	1	1
Translocation + inversion	0	3	3
3 patterns identified	0	1	1
5′ deletion + translocation + inversion	0	1	1

**Table 5:** *ALK* discordant and concordant cases analyzed with NGS.

Case No.	Gender	Age	ALK FISH Pattern	ALK Copy Number Gains	IHC	NGS
1.	M	69	rearranged nuclei: 26% pattern: translocation + inversion	16% 4–5 copies; 1% 9 copies	negative	IA: no variants reported; IB: *TP53* p.P190L c.569C>T
2.	M	60	rearranged nuclei: 68% pattern: inversion	17% 4–10 copies; 8% >10 copies	negative	IA: no variants reported; IB: no variants reported
3.	M	53	rearranged nuclei: 64% pattern: inversion	not detected	negative	IA: *EML4, ALK* fusion transcript; IB: *MYC* copy number gain (3 copies)
4.	M	70	rearranged nuclei: 44% pattern: 5′ deletion + translocation	11% 4–6 copies	negative	IA: no variants reported; IB: *EGFR* copy number gain (10 copies) *MYC* copy number gain (3 copies) TP53 p.I195T c.584T>C
5.	M	68	rearranged nuclei: 30% pattern: translocation + interstitial deletion	52% 4–10 copies	negative in limited material	IA: no variants reported; IB: *MET* copy number gain (8 copies) *EGFR* copy number gain (3 copies) *BRAF* copy number gain (5 copies) *MYC* copy number gain (3 copies)
6.	F	70	adenocarcinoma component: rearranged nuclei: 26% pattern: inversion squamous cell carcinoma component: rearranged nuclei: 6% *ALK* negative	Adenocarcinoma component: 14% 4–7 copies Squamous cell carcinoma component: not detected	negative	IA: no variants reported; IB: no variants reported
7.	M	64	rearranged nuclei: 18% pattern: 5′ deletion + inversion	not detected	negative	IA: no variants reported; IB: *TP53* p.P152Rfs
8.	F	75	rearranged nuclei: 17% pattern: 5′ deletion + translocation + inversion	13% 4 copies; 12% 5–6 copies; 1% 7–8 copies; 5% more than 10 copies	negative	IA: no variants reported; IB: *MYC* copy number gain (22 copies)
9.	M	78	rearranged nuclei: 60% pattern: 5′ deletion	4% 4 copies; 4% 5 copies	negative	IA: no variants reported; IB: no variants reported
10.	M	63	moderately differentiated component: rearranged nuclei: 4% *ALK* negative poorly differentiated component: rearranged nuclei: 16% pattern: translocation + inversion	Moderately differentiated component: 22% 4 copies; 18% 5–8 copies Poorly differentiated component: 10% 4 copies; 2% 5–6 copies	negative	IA: no variants reported; IB: no variants reported
11.	F	79	rearranged nuclei: 4% *ALK* negative (38% positive in analysis with the break apart probe; population not found in further sections)	4% 4 copies; 2% 7–8 copies	Negative, 100 positive nuclei	IA: no variants reported; IB: no variants reported
12.	F	77	rearranged nuclei: 4% *ALK* negative (according to valid guidelines) abnormal signal configuration: - 28% isolated 5′ green signal co-localizing with blue signal, isolated blue signal - 32% isolated 5′ green signal co-localizing with blue signal - 14% isolated 5′ green signal co-localizing with blue signals, isolated blue signal; another copy isolated 5′ green signal co-localizing with blue signal	not detected	positive	IA: *EML4, ALK* fusion transcript IB: no variants reported
13.	F	71	ALK/EML4 probe not used rearranged nuclei: 2% using the ALK break apart probe - 1% isolated 3′ orange signal, isolated 5′ green signal - 1% isolated 3′ orange signal	not detected	positive	IA: *CSFT3, ALK* fusion transcript IB: no variants reported
14.	F	56	rearranged nuclei: 50% pattern: inversion	not detected	positive	IA: *EML4, ALK* fusion transcript IB: no variants reported
15.	F	73	rearranged nuclei: 48% pattern: interstitial deletion + inversion	not detected	positive	IA: *EML4, ALK* fusion transcript IB: EGFR copy number gain (3 copies)
16.	M	48	rearranged nuclei: 62% pattern: 5′ deletion + interstitial deletion	not detected	positive	IA: *HIP1, ALK* fusion transcript IB: no variants reported
17.	F	64	rearranged nuclei: 90% pattern: interstitial deletion	positive nuclei display up to 4 rearranged signals	positive	IA: *EML4, ALK* fusion transcript IB: no variants reported

## Data Availability

The data presented in this study are available on request from the corresponding author.

## Supplementary Materials

The following supporting information can be downloaded at https://www.mdpi.com/article/10.3390/ijms25158168/s1.
