# Supplementary material for: Discordant ALK Status in Non-Small Cell Lung Carcinoma: A Detailed Reevaluation Comparing IHC, FISH, and NGS Analyses
Source: Int J Mol Sci. 2024 Jul 26;25(15):8168. doi: 10.3390/ijms25158168 (PMC11312000; doi:10.3390/ijms25158168)
Supplement: Supplementary file 1 [file ijms-25-08168-s001.zip › Supplementary_Material_1_Statistical_Analysis.pdf]

Supplementary Material 1: Statistical analysis

Q1

	concordant	discordant
**1 pattern**	137	12
**2 patterns**	30	14
**3 patterns**	1	3

Mosaicplot

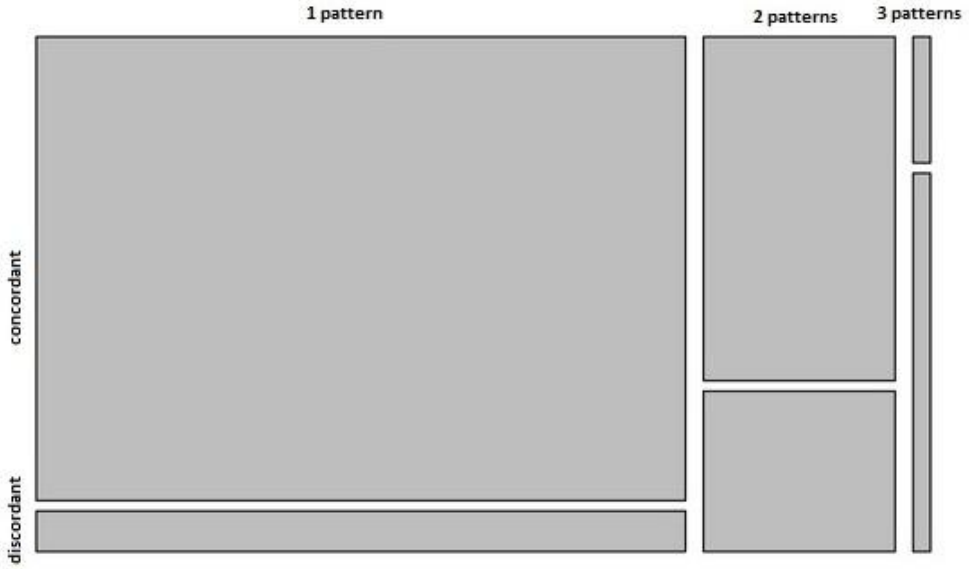

Fisher's exact test

Fisher's Exact Test for Count Data
data: ta
p-value = 0.000005526
alternative hypothesis: two sided

Cramer's V with 95 % CI

Two-sided 95% chi-squared confidence interval for the population Cramer's V

Sample estimate: 0.3708887
Confidence interval:
2.5% 97.5%
0.2441778 0.5146194

Results

There is a statistically significant association (p-value 0.0000055) between the type of results (concordant/discordant) and variant of FISH signal (three patterns). Using the standard calibration of

Cramer's V for 2 degrees of freedom, the association may be judged strong (Cramer's V is 0.37, with 95% confidence interval (0.24, 0.51).

Q2

	concordant	discordant
**Inversion in all combinations**	158	22
- **single pattern**	133	10

Mosaicplot

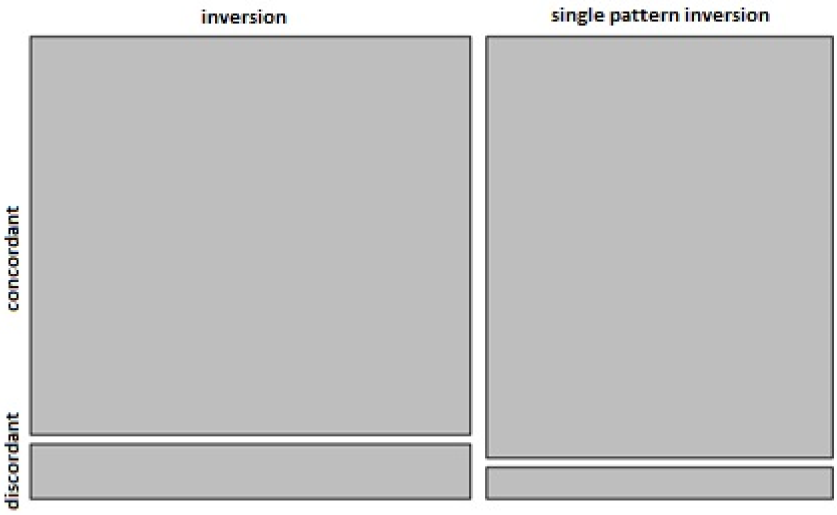

Fisher's exact test

Fisher's Exact Test for Count Data
data: ta
p-value = 0.1376
alternative hypothesis: true odds ratio is not equal to 1
95 percent confidence interval:
0.2233043 1.2564106
sample estimates:
odds ratio
0.5477975

Cramer's V with 95 % CI

Two-sided 95% chi-squared confidence interval for the population Cramer's V

Sample estimate: 0.08488501
Confidence interval:
2.5% 97.5%
0.0000000 0.2013938

Q3

	concordant	discordant
**Interstitial deletion in all combinations**	31	4
- **single pattern**	4	0

Mosaicplot

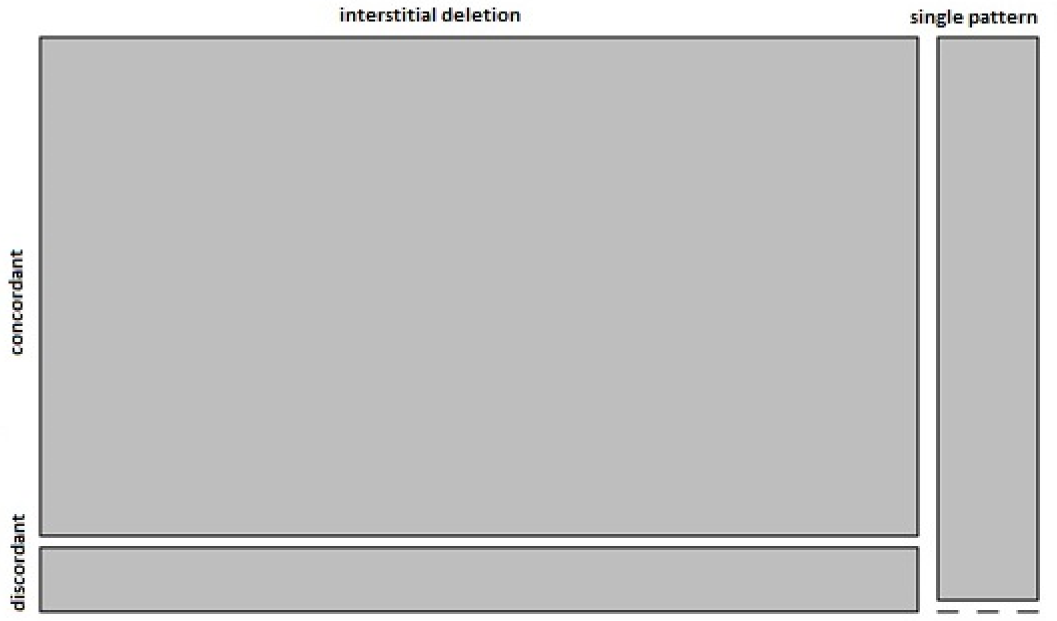

Fisher's exact test

Fisher's Exact Test for Count Data
data: ta
p-value = 1
alternative hypothesis: true odds ratio is not equal to 1
95 percent confidence interval:
0.00000 16.27047
sample estimates:
odds ratio
0

Cramer's V with 95 % CI

Two-sided 95% chi-squared confidence interval for the population Cramer's V

Sample estimate: 0.1142857
Confidence interval:
2.5% 97.5%
0.000000 0.456177

Q4

	concordant	discordant
**5′ deletion in all combinations**	5	11
- **single pattern**	0	2

Mosaicplot

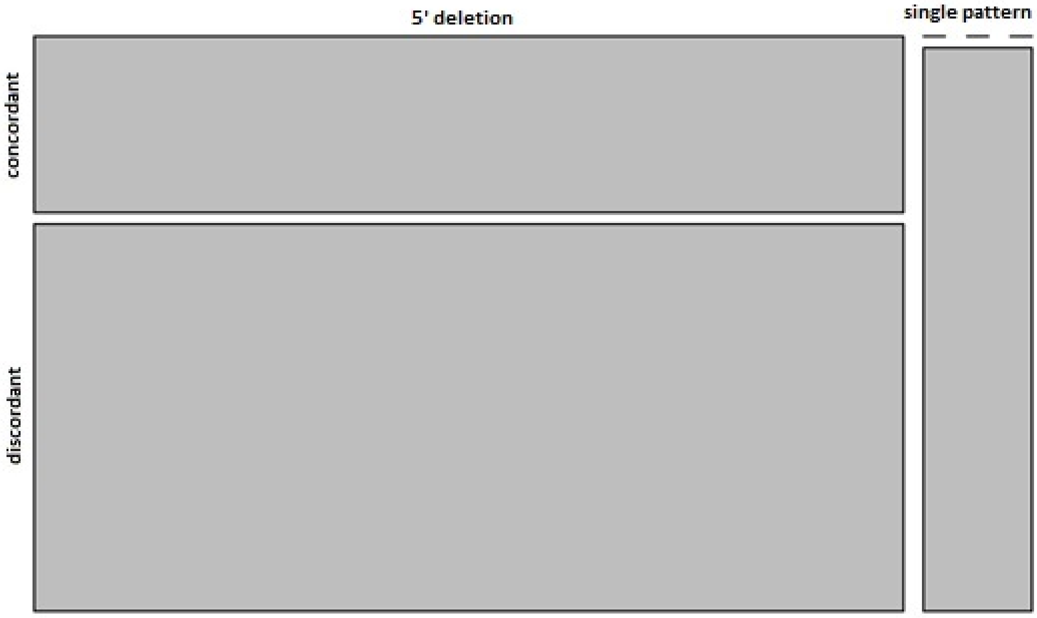

Fisher's exact test

Fisher's Exact Test for Count Data

data: ta
p-value = 1
alternative hypothesis: true odds ratio is not equal to 1
95 percent confidence interval:
0.06900251 Inf
sample estimates:
odds ratio
Inf

Cramer's V with 95 % CI

Two-sided 95% chi-squared confidence interval for the population Cramer's V

Sample estimate: 0.2192645
Confidence interval:
2.5% 97.5%
0.0000000 0.7206005

Q5

	concordant	discordant
**translocation in all combinations**	6	12
- **single pattern**	0	0

Mosaicplot

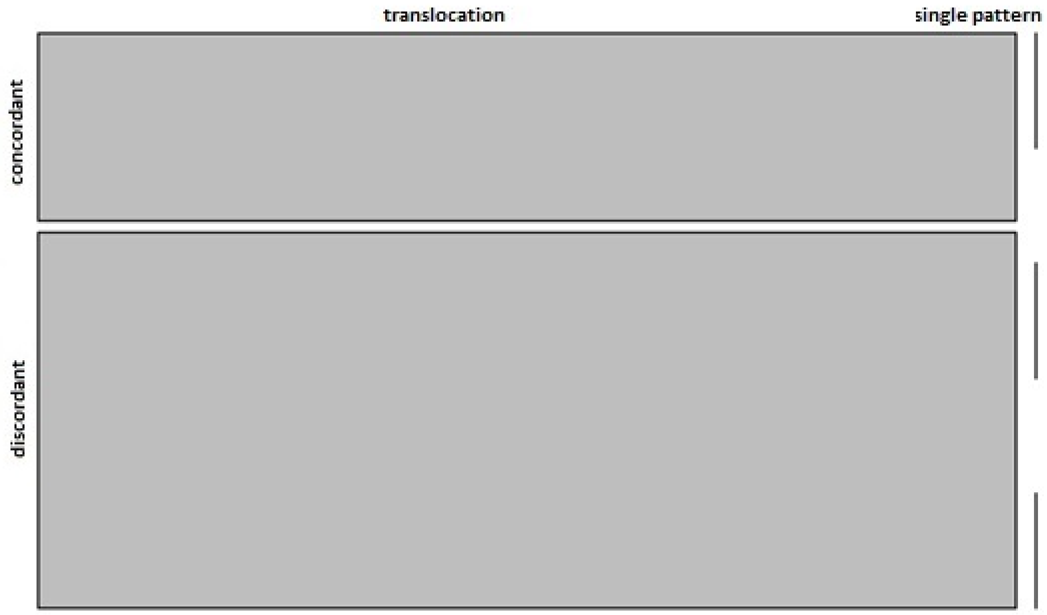

Fisher's exact test

Fisher's Exact Test for Count Data

data: ta
p-value = 1
alternative hypothesis: true odds ratio is not equal to 1
95 percent confidence interval:
0 Inf
sample estimates:
odds ratio
0

Cramer's V with 95 % CI

Two-sided 95% chi-squared confidence interval for the population Cramer's V

Sample estimate: NaN
Confidence interval:
2.5% 97.5%
0 1

Laplace smoothing (add 1 to table)

Fisher's exact test

Fisher's Exact Test for Count Data

data: tat
p-value = 1
alternative hypothesis: true odds ratio is not equal to 1
95 percent confidence interval:
 0.006386058 48.249114710
sample estimates:
odds ratio
 0.5546792

Cramer's V with 95 % CI

Two-sided 95% chi-squared confidence interval for the population Cramer's V

Sample estimate: NaN
Confidence interval:
 2.5% 97.5%
 0 1
