# Supplementary material for: Discordant ALK Status in Non-Small Cell Lung Carcinoma: A Detailed Reevaluation Comparing IHC, FISH, and NGS Analyses
Source: Int J Mol Sci. 2024 Jul 26;25(15):8168. doi: 10.3390/ijms25158168 (PMC11312000; doi:10.3390/ijms25158168)
Supplement: Supplementary file 1 [file ijms-25-08168-s001.zip › Supplementary_Material_2.pdf]

Supplementary Material 2: Examples of ALK/EML4 assortment performed according to the scheme listed as Figure 3

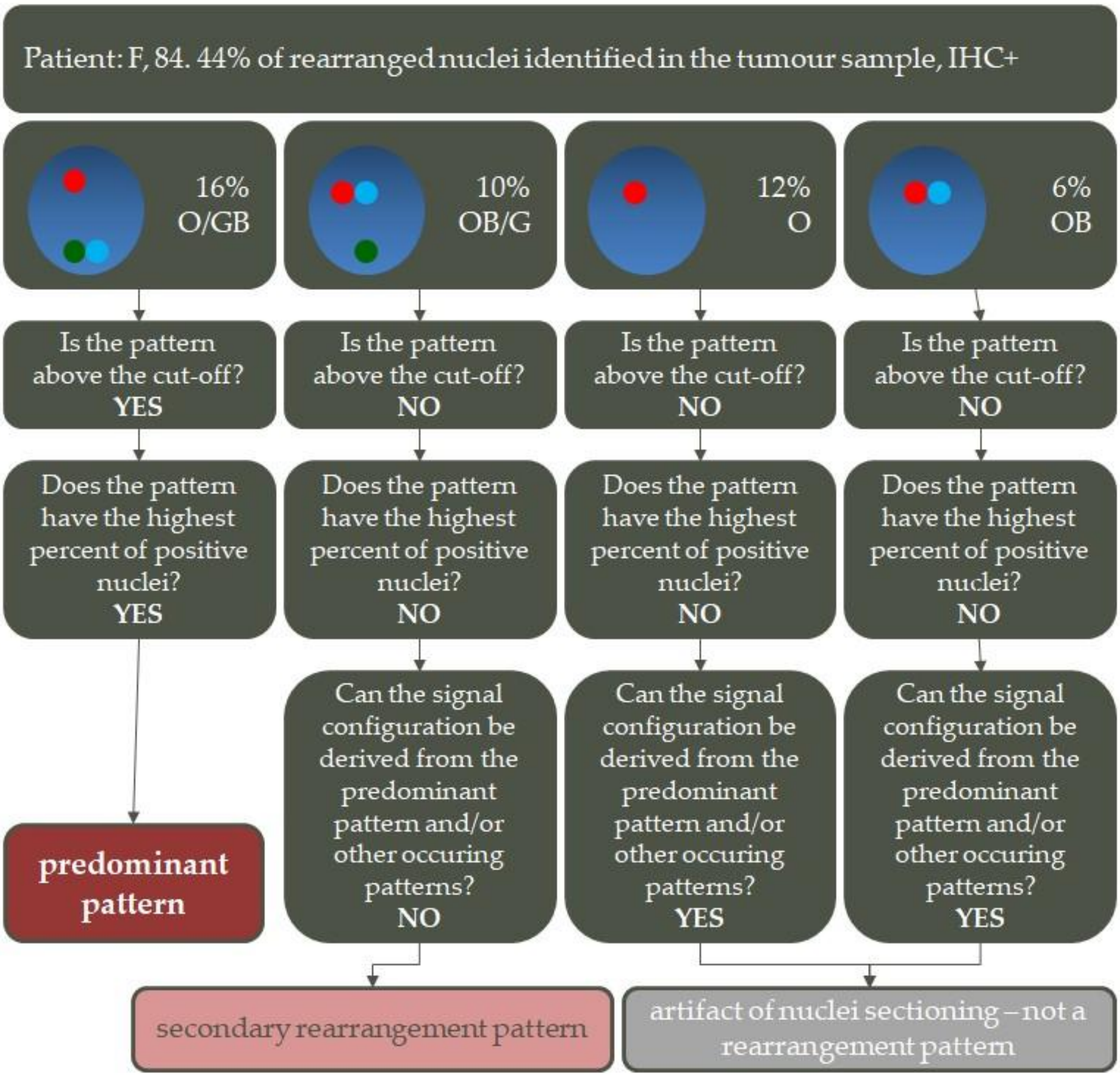

a) Female, 84 years old at the time of diagnosis, *ALK* positive concordant. (FISH result: *ALK* locus is rearranged in 44% of evaluated nuclei; positive ALK protein expression), evaluated as featuring translocation as the main pattern and inversion as the secondary pattern.

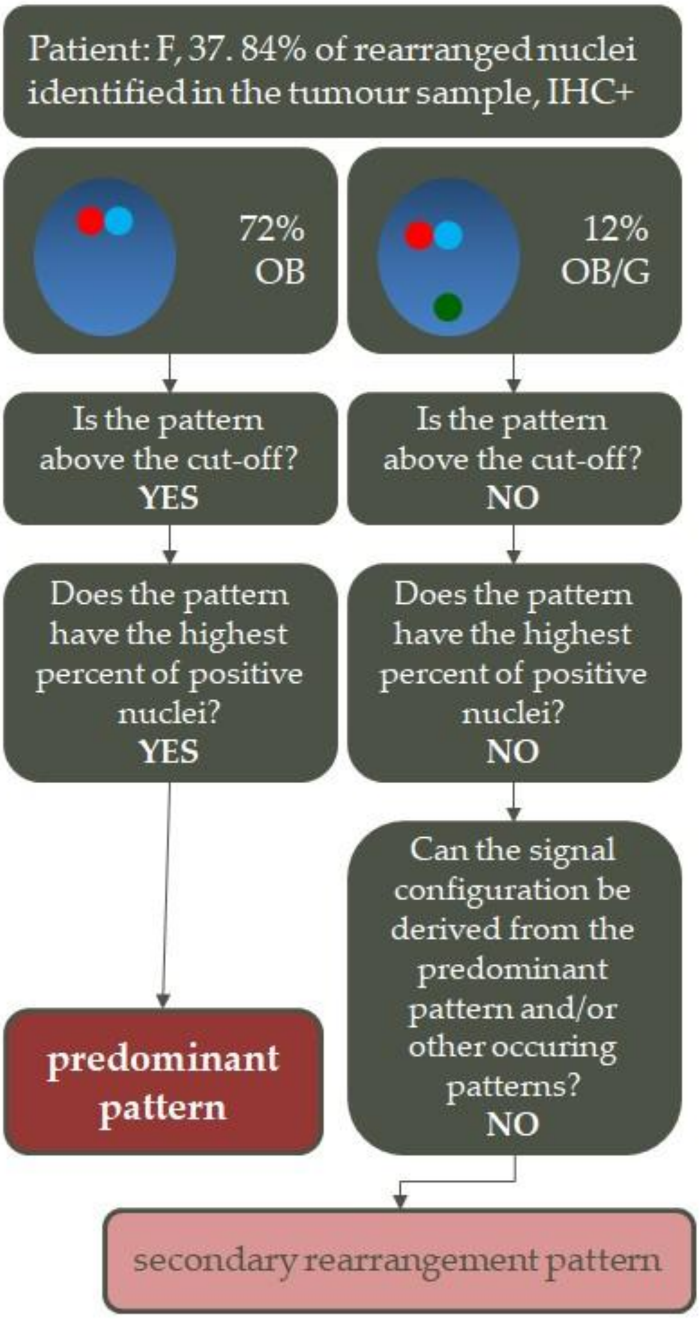

b) Female, 37 years old at the time of diagnosis, *ALK* positive concordant (FISH result: *ALK* locus is rearranged in 84% of evaluated nuclei, positive ALK protein expression), evaluated as featuring interstitial deletion as the main pattern and translocation as the secondary pattern.

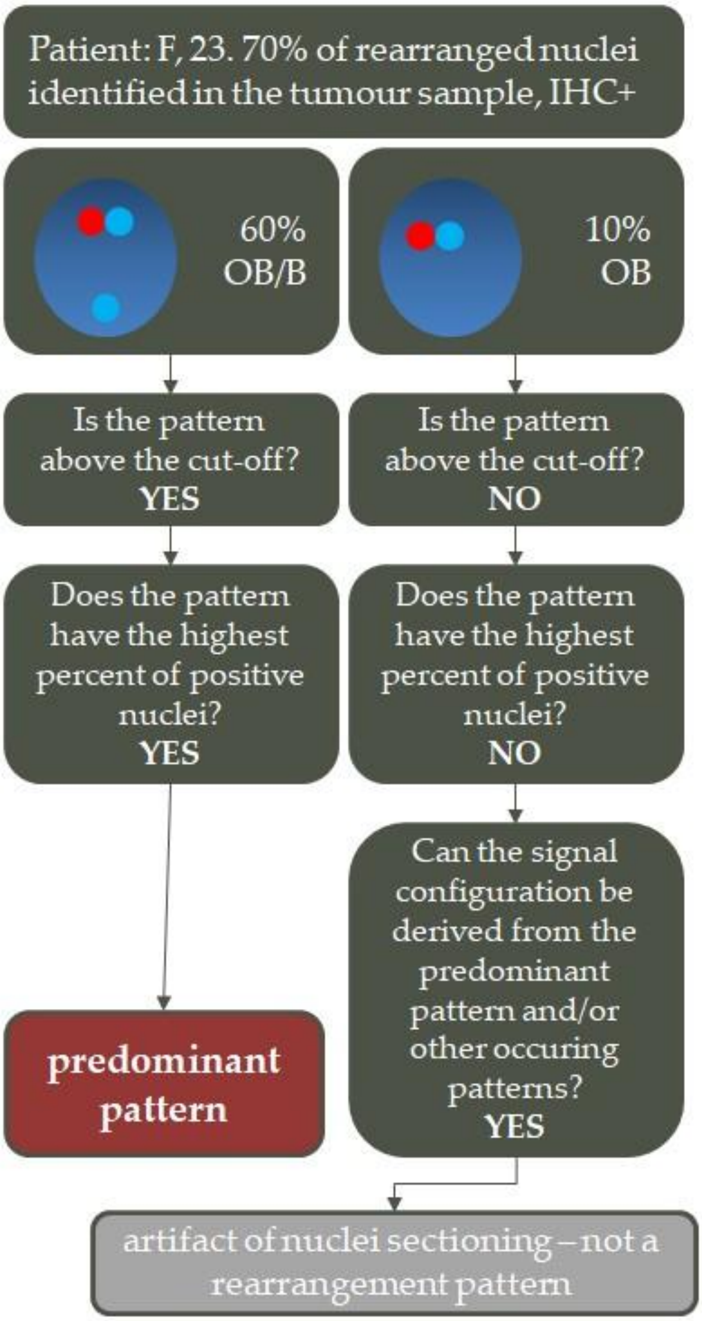

c) Female, 23 years old at the time of diagnosis, *ALK* positive concordant (FISH result: *ALK* locus is rearranged in 70% of evaluated nuclei, positive ALK protein expression), evaluated as featuring inversion as the single pattern of rearrangement.

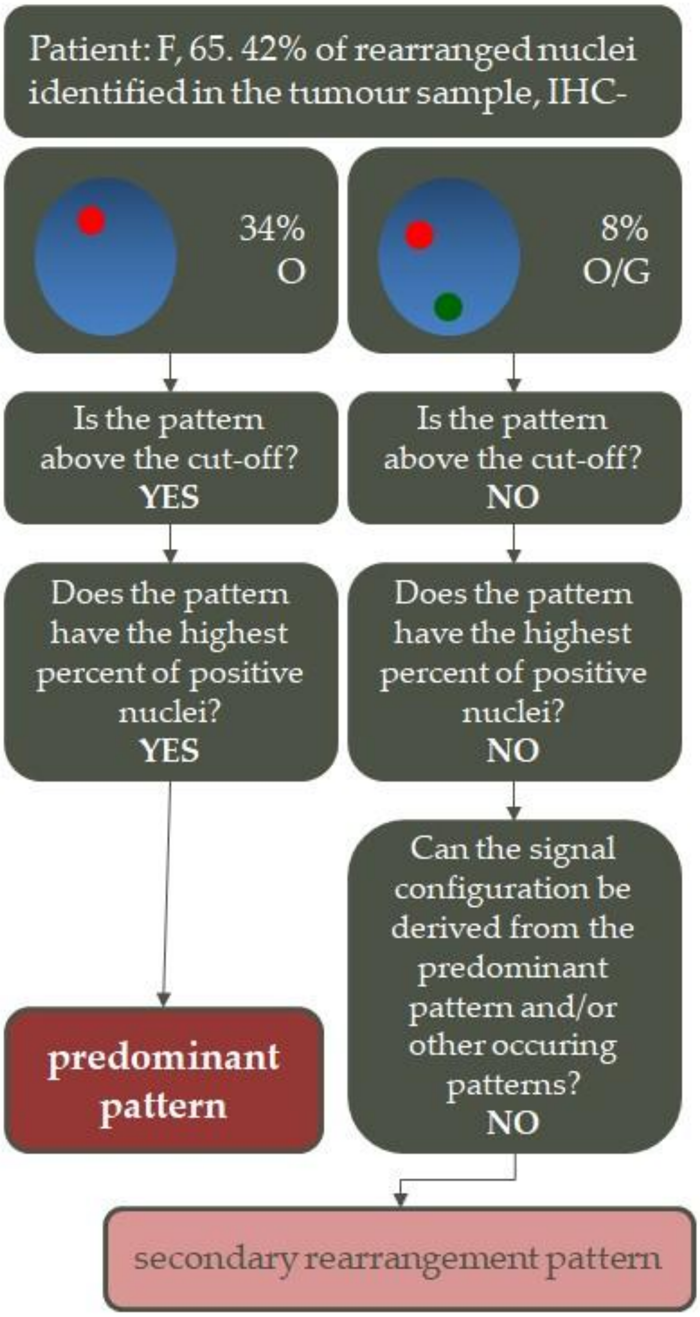

d) Female, 65 years old at the time of diagnosis, *ALK* positive discordant (FISH result: *ALK* locus is rearranged in 42% of evaluated nuclei, negative ALK protein expression), evaluated as featuring 5′ deletion as the main pattern and translocation as secondary pattern of rearrangement.

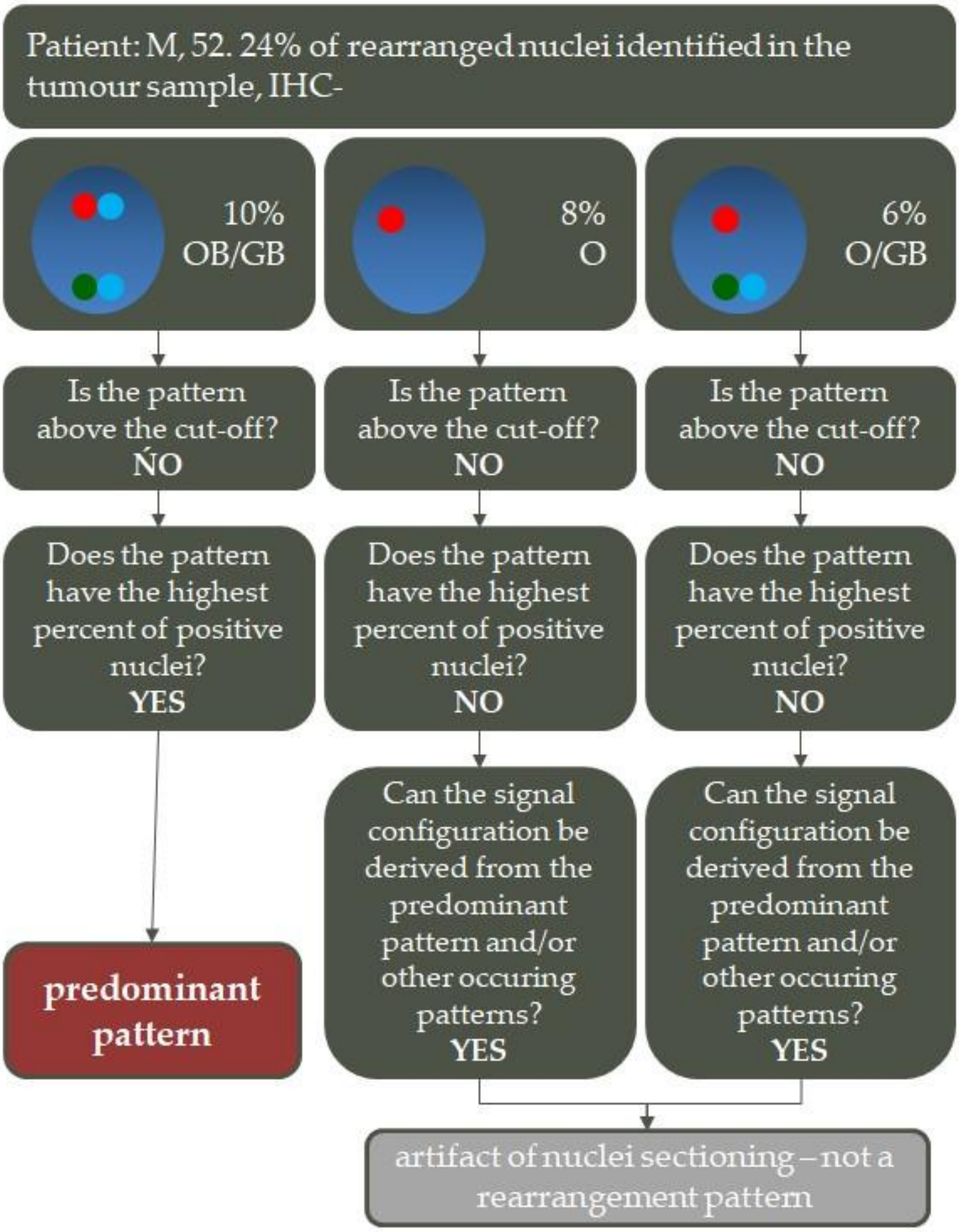

e) Male, 52 years old at the time of diagnosis, *ALK* positive discordant (FISH result: *ALK* locus is rearranged in 24% of evaluated nuclei, negative ALK protein expression), evaluated as featuring inversion as the main pattern of rearrangement.
